# Current status and risk determinants of locomotive syndrome in geriatric cancer survivors in China—a single-center cross-sectional survey

**DOI:** 10.3389/fpubh.2024.1421280

**Published:** 2024-11-29

**Authors:** Yu-Ling Yang, Hui Su, Hui Lu, Hui Yu, Jing Wang, Yu-Qing Zhou, Ling Li, Ying Chen

**Affiliations:** Affiliated Hospital of Jiangnan University, Wuxi, Jiangsu, China

**Keywords:** geriatric cancer survivors, locomotive syndrome, prevalence, risk determinants, motor function

## Abstract

**Objective:**

To assess the prevalence and risk determinants of locomotive syndrome (LS) in geriatric cancer survivors in China. To generate evidence-based insights for the clinical prevention and intervention strategies concerning LS in this vulnerable population, emphasizing the need for integrated public health initiatives focused on maintaining mobility among geriatric cancer survivors.

**Methods:**

Six hundred geriatric cancer survivors were recruited at a hospital in China. A demographic questionnaire, the International Physical Activity Questionnaire-Short (IPAQ-S), and the Geriatric Locomotive Function Scale (GLFS-25) were administered. Survivors were stratified into three physical activity level (PAL) groups via IPAQ-S scores: low, medium, and high. LS was operationally defined via GLFS-25 scores, with cut-offs established for LS-1, LS-2, and LS-3. Elevated GLFS-25 scores signified deteriorated motor function (MF) and increased severity of LS. Data analysis was done to investigate the risk determinants to the occurrence and exacerbation of LS among geriatric cancer survivors.

**Results:**

Of the 524 geriatric cancer survivors who completed the study, 292 (55.7%) were diagnosed with LS, including 152 (29%) categorized under LS-1, 52 (9.9%) under LS-2, and 88 (16.8%) under LS-3. Univariate analysis indicated that variations in exercise habits, prior occupational type, presence of tumor metastasis or recurrence, visual impairments, somatosensory abnormalities, and PAL were significantly associated with differing occurrences and severities of LS (*p* < 0.05). Ordinal logistic regression revealed that prior occupational type (OR = 0.466), tumor metastasis (OR = 0.404), tumor recurrence (OR = 0.341), and PAL (medium: OR = 7.178; high: OR = 1.984) were independent risk determinants modulating both the occurrence and severity of LS in cancer survivors (*p* < 0.05).

**Conclusion:**

The occurrence of LS is notably elevated among geriatric cancer survivors in China, indicating a significant public health concern. Individuals who were previously engaged in non-physically demanding occupations and those with histories of tumor metastasis or recurrence, coupled with reduced PAL, demonstrate a heightened susceptibility and severities to LS. Early identification of these risk determinants is imperative for mitigating the onset and progression of LS. Comprehensive public health strategies, including regular screening programs, targeted physical rehabilitation initiatives, and community-based interventions, are essential to mitigate the onset and progression of LS in this vulnerable population, ultimately reducing its broader impact on aging-related health outcomes.

## 1 Introduction

A cancer survivor is a person who has survived cancer for a long time. The European Organization for Research and Treatment of Cancer (EORTC) defines a cancer survivor as an individual diagnosed with a malignant tumor who has completed primary antitumor therapy, has no evidence of an active tumor, and is in long-term remission (1, 2). Based on the 5-year survival rate, some studies have defined tumor patients who have survived for more than 5 years without an active tumor after a diagnosis of malignancy as tumor survivors (3). Some of these long-term survivors are actually clinically cured and are not suitable to be called “patients,” but they are still cancer survivors.

As tumor treatments progress, more and more tumor survivors are returning to society to continue their lives. According to the latest statistics in 2016, there are about 15.5 million cancer survivors in the United States, and this number is expected to increase to 26.1 million in 2040 (4). In contrast, data from China shows that in 2011 the number of cases in China that were diagnosed within 5 years and survived without active tumors was 7.49 million, with new patients aged 60–79 years (5, 6). This group will expand dramatically in the coming decades with the changes in social life patterns and the development of medical technology (6). However, after the initial intense anti-cancer treatment, these survivors continue to suffer from a variety of tumors and long-term sequelae from the anti-tumor treatments, many of which are psychologically and physically difficult. Enhancing survivors’ quality of life (QoL) through public health strategies is a crucial component of modern oncology, alongside improving survival outcomes. Central to improving QoL is the preservation of survivors’ motor function (MF), which is fundamental to maintaining essential activities of daily living (ADL) (7).

In order to accentuate public awareness about the cardinality of MF and ADL, the Japanese Orthopaedic Association (JOA) promulgated the concept of locomotive syndrome (LS)—a state characterized by the degradation or functional incapacitation of locomotor organs (8). The progression of LS culminates in functional constraints on limb activities, eventually compromising MF to the point of necessitating external care or causing bedridden states (8). Empirical research substantiates that LS is intrinsically linked with adverse public health outcomes such as diminished ADL, increased susceptibility to fractures, and elevated mortality rates (9). The global clinical and research focus on LS underscores its public health relevance, particularly given the growing aging population and cancer survivors (10).

Unlike sarcopenia or muscular weakness, LS provides a more holistic assessment of the body’s overall MF (10). The insidious degradation of locomotor constituents, notably the musculoskeletal system, often eluding early detection. Among cancer survivors whose Eastern Cooperative Oncology Group Performance Status (ECOG-PS) denoted a “Fully Active” categorization, 29.7% were diagnosed with LS, even in the absence of overt sarcopenia or muscular weakness (11). Masahiro contends that LS serves as a more sensitive barometer for gauging MF impairment, and its timely diagnosis facilitates preemptive interventions, thereby preserving ADL and QoL (12).

Vulnerability to LS is notably pronounced among geriatric cancer survivors. A range of bodily functions decline as a result of aging. Also, oncological afflictions can necessitate prolonged bedridden states, subsequently inducing muscle disuse atrophy, secondary osteoporosis, and radiation or surgery-induced lymphedema, etc.—each serving as a potential catalyst for LS development (12, 13). Early detection and intervention strategies to address LS within the geriatric cancer survivor population offer a significant opportunity for public health programs aimed at sustaining or improving ADL and QoL. The objective of this study is to explore the prevalence and risk factors associated with LS among geriatric cancer survivors, offering a foundation for developing targeted public health strategies to prevent and manage this condition.

## 2 Materials and methods

This investigation employed a single-center, cross-sectional study design. This study employed a cross-sectional study design. Participant voluntarily participated in the study and signed the consent. The study was approved by the Ethics Committee of the Affiliated Hospital of Jiangnan University (No. LS2023101) and completed the Chinese Clinical Trial Registration (No. ChiCTR2400079958), the date of first registration is 17/01/2024. All participants provided written informed consent. This study conformed to the standards of the Declaration of Helsinki.

### 2.1 Subjects

The recruitment of geriatric cancer survivors was executed through a convenience sampling methodology in the oncology ward of a Grade-A hospital in China, spanning from January to March 2024, at the Cancer Center of the Affiliated Hospital of Jiangnan University, Wuxi, Jiangsu Province, China. Individuals were selected from those attending outpatient appointments.

Inclusion Criteria: ① Participants must have a definitive cancer diagnosis. Currently defined no active tumor. ② Completion of therapeutic interventions including surgery, radiotherapy, and chemotherapy (active treatment, not palliative care). Survival of ≥5 years without active tumor. ③ Age > 60 years old. ④ Presence of stable vital signs. ⑤ Cognitive clarity and coherent responsiveness. ⑥ Capability for autonomous self-care. ⑦ Absence of any MF disorder attributable to primary orthopedic diseases. ⑧ Provision of informed consent and voluntary participation in the study.

Exclusion Criteria: ① Cognitive impairment or unconsciousness. ② Diagnosis of any incapacitating ailment contraindicating physical activity (PA).

### 2.2 Measurements

A structured questionnaire was fine-tuned based on insights from exhaustive literature review, clinical experts and pre-existing validated scales. The survey incorporated five primary domains:

1. Sociodemographic Data: Including gender, age, height, weight, ADL, etc.
2. Disease-specific Variables: Information about participants when they had active tumors in the past, including tumor type, metastatic occurrences, tumor recurrence, therapeutic modalities of tumors, whether they have visual abnormalities, auditory abnormality, somatosensory abnormality that significantly affect their daily lives, etc.
3. Body Mass Index, BMI: BMI = weight/height^2^ in international units of kg/m^2^. The current Chinese adult BMI classification standard is four levels: <18.5 is underweight, 18.5–23.9 is normal, 24.0–27.9 is overweight, and ≥ 28.0 is obesity (14).
4. PA: The International Physical Activity Questionnaire-Short Form (IPAQ-S) was employed. The IPAQ-S prompts participants to retrospectively catalog the frequency, duration, and sedentary behavior pertaining to PA engaged in over the prior one-week period. Subsequently, researchers calculated the aggregate physical activity level (PAL), expressed in Metabolic Equivalent of Task minutes per week (MET-min/week), based on the MET values attributed to diverse exercises (15).

Operational definitions for categorizing PAL:

- High: Involvement in high-intensity PA for a minimum of 3 days, accumulating at least 1,500 MET-min/week, or engagement in PA of any intensity for 7 days, accruing at least 3,000 MET-min/week.
- Medium: Participation in high-intensity PA for at least 3 days, each consisting of a 20-min session; or moderate-intensity PA and/or walking for a minimum of 5 days, with each session lasting at least 30 min; or engagement in PA of various intensities for at least 5 days, amassing a minimum of 600 MET-min/week.
- Low: Either no reported PA or involvement in some PA that failed to meet the criteria for the medium or high PAL categories.

5. Tests and definition of LS: Developed by experts with extensive clinical acumen at the JOA, the Geriatric Locomotive Function Scale (GLFS-25) serves as a nuanced, quantitative, evidence-based instrument specifically designed for the early detection of LS (16). The GLFS-25 incorporates a composite of 25 items. Employing a Likert scale, ranging from “No difficulty” (scored as 0) to “Extremely difficult” (scored as 4), the scale’s cumulative scores extend from 0 to 100 points. LS was operationalized as a total score of ≥7, with higher scores signifying diminished MF and increased severity of LS. According to JOA-established criteria, three gradations of LS were identified: LS-1 (GLFS-25 score: 7–15), LS-2 (GLFS-25 score: 16–23), and LS-3 (GLFS-25 score: 24–100) (7, 11). LS-1 signifies incipient motor function degradation; LS-2 represents advanced MF impairment with heightened risk for compromised independent living; LS-3 indicates severe MF dysfunction (11). The psychometric properties of the GLFS-25 exhibited high reliability and validity, with a Cronbach’s α coefficient of 0.961 (16).

### 2.3 Sample size

Based on methodological convention, the sample size was ascertained to be quintuple the number of variables incorporated into the GLFS-25. At least 10% attrition rate was judiciously accounted. Consequently, a cohort of 600 cancer survivors was deemed adequate and participated in the study.

### 2.4 Data collection

This study adhered to rigorous protocols to ensure anonymity and the confidentiality of participant data. Prior to questionnaire administration, investigators elucidated the individual items to participants and addressed any queries to clarify their understanding of the instrument. The respondents completed the questionnaire under supervised conditions, thereby enhancing the veracity and objectivity of the collected data. Upon immediate collection, the completed questionnaires underwent meticulous scrutiny and double verification to ascertain their comprehensiveness and accuracy.

### 2.5 Statistical methods

The analysis of the collected data was conducted utilizing SPSS version 24.0. For continuous data adhering to a normal distribution, metrics were articulated as the mean ± standard deviation (Mean ± SD). Data demonstrating a skewed distribution were depicted through the median along with the interquartile range. Categorical variables were characterized by both frequency and percentage. Univariate analytical procedures were executed employing either the Wilcoxon rank-sum test or the Kruskal-Wallis H test. Specifically, these univariate analyses featured the LS gradation in cancer survivors as the dependent variable, with all survey items serving as independent variables. Variables that manifested statistically significant disparities in the univariate analyses were subsequently incorporated into an ordinal logistic regression model. This was designed to elucidate the independent risk determinants associated with the manifestation of LS among the cancer survivors. *p*-value less than 0.05 was established as the threshold for statistical significance.

## 3 Results

### 3.1 General information of Chinese geriatric cancer survivors, the occurrence and severity of LS

**Figure 1:**
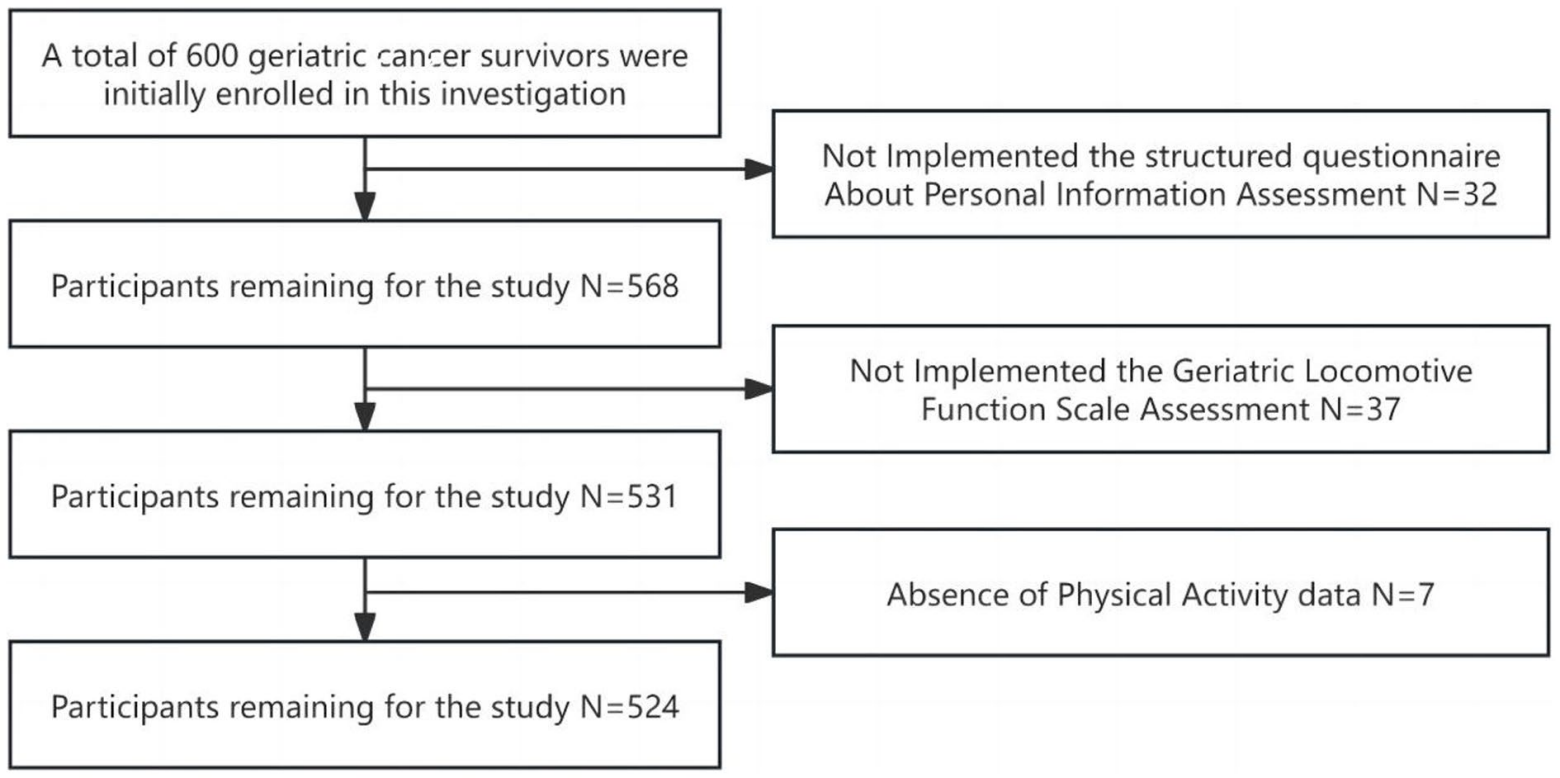
Flow chart of study participants.

A comprehensive cohort of 600 geriatric cancer survivors were initially enrolled in this investigation. Following the exclusion of incomplete questionnaires, 524 subjects yielded analyzable data, reflecting a response rate of 87.3% (Figure 1). The mean age of participants spanned 65 years old with an interquartile range of 60–89 years old, and the median duration of disease stood at 8 years, with an interquartile range of 5–20 years. The diagnostic distribution was as follows: gastrointestinal tumors comprised 35.1% (184 cases); lung tumors 17.6% (92 cases); head and neck tumors 6.8% (36 cases); breast tumors 18.3% (96 cases); liver tumors 4.6% (24 cases); tumors of the reproductive system 15.3% (80 cases); and hematological malignancies 2.3% (12 cases). A preponderance of subjects, 66.4% (348 cases), did not present any co-morbid chronic disease (including hypertension, diabetes mellitus, dyslipidemia, stroke and chronic respiratory diseases), while 32.1% (168 cases) had one to two additional chronic disease, and 1.5% (8 cases) reported three or more. The median BMI for participants was 22.76, with an interquartile range of 20.31 to 24.22, conforming to normal weight criteria. The median total PAL was quantified as 495 MET-min/week, which classified the cohort predominantly in the low PAL category. The mean GLFS-25 score was 8 with an interquartile range of 2 to 16. LS was observed in 55.7% (292 cases) of the participants: 52.1% (152 cases) were categorized as LS-1, 17.8% (52 cases) as LS-2, and 30.1% (88 cases) as LS-3. Detailed data are presented in Table 1.

**Table 1:** Comparison of the occurrence and severity of LS in cancer survivors with different characteristics.

Items	Groups	Total (%)	Non−LS (%)	LS−1 (%)	LS−2 (%)	LS−3 (%)	Mean rank	Z/H	*p*
Age (Years)	60~69	292 (55.7)	128 (43.8)	76 (26.0)	28 (9.6)	60 (20.6)	67.74	0.751^2^	0.687
	70~79	176 (33.6)	80 (45.5)	60 (34.1)	16 (9.1)	20 (11.3)	61.10		
	80~	56 (10.7)	24 (42.9)	16 (28.5)	8 (14.3)	8 (14.3)	66.71		
Gender	Male	252 (48.1)	108 (42.9)	68 (27.0)	32 (12.6)	44 (17.5)	67.67	−0.517^1^	0.605
	Female	272 (51.9)	124 (45.6)	84 (30.9)	20 (7.4)	44 (16.2)	64.45		
BMI	Underweight	48 (9.2)	20 (41.7)	12 (25.0)	8 (16.7)	8 (16.7)	68.92	0.121^2^	0.989
	Normal	320 (61.1)	140 (43.8)	104 (32.5)	24 (7.5)	52 (16.2)	65.40		
	Overweight	116 (22.1)	56 (48.3)	20 (17.2)	16 (13.8)	24 (20.7)	66.74		
	Obesity	40 (7.6)	16 (40.0)	16 (40.0)	4 (10.0)	4 (10.0)	65.15		
Exercise habit	None	344 (65.6)	144 (41.9)	92 (26.7)	48 (14.0)	60 (17.4)	67.94	−2.430^1^	0.015^*^
	Yes	180 (34.4)	88 (48.9)	60 (33.3)	4 (2.2)	28 (15.6)	52.10		
Type of former occupation	Physical Labor	184 (35.1)	68 (37.0)	36 (20.0)	28 (15.2)	52 (28.3)	75.79	−2.312^1^	0.021^*^
	Non−physical Labor	340 (64.9)	164 (48.2)	116 (34.1)	24 (7.1)	36 (10.6)	60.70		
Tumor metastasis	None	268 (51.1)	156 (58.2)	68 (25.4)	20 (7.5)	24 (9.0)	55.31	−3.508^1^	0.000^*^
	Yes	256 (48.9)	76 (29.7)	84 (32.8)	32 (12.5)	64 (25.0)	77.19		
Operation	None	180 (34.4)	60 (33.3)	60 (33.3)	12 (6.7)	48 (26.7)	74.87	−1.530^1^	0.126
	Yes	344 (65.6)	172 (50.0)	92 (26.7)	40 (11.6)	40 (11.6)	64.57		
Radiotherapy	None	408 (77.9)	180 (44.1)	132 (32.4)	32 (7.8)	64 (15.7)	65.07	−0.560^1^	0.575
	Yes	116 (22.1)	52 (44.8)	20 (17.2)	20 (17.2)	24 (20.7)	69.28		
Chemotherapy	None	100 (19.1)	36 (36.0)	40 (40.0)	16 (16.0)	8 (8.0)	67.74	−0.271^1^	0.786
	Yes	424 (80.9)	196 (46.2)	112 (26.4)	36 (8.5)	80 (18.9)	65.59		
Recurrence	None	436 (83.2)	212 (48.6)	136 (31.2)	32 (7.3)	56 (12.8)	61.56	−3.174^1^	0.002^*^
	Yes	88 (16.8)	20 (22.7)	16 (18.2)	20 (22.7)	32 (36.4)	88.02		
History of falling	None	500 (95.4)	228 (45.6)	136 (27.2)	52 (10.4)	84 (16.8)	65.49	−0.750^1^	0.453
	Yes	24 (4.6)	4 (16.7)	16 (66.7)	0 (0.0)	4 (16.7)	76.67		
Visual abnormality	None	476 (90.8)	224 (47.1)	136 (28.6)	44 (9.2)	72 (15.1)	63.77	−2.250^1^	0.024^*^
	Yes	48 (9.2)	8 (16.7)	16 (33.3)	8 (16.7)	16 (33.3)	88.08		
Auditory abnormality	None	496 (94.7)	224 (45.2)	140 (28.2)	48 (9.7)	84 (16.9)	65.57	−0.577^1^	0.564
	Yes	28 (5.3)	8 (28.6)	12 (42.9)	4 (14.3)	4 (14.3)	73.57		
Somatosensory abnormality	None	440 (84)	216 (49.1)	116 (26.4)	36 (8.2)	72 (16.4)	63.06	−2.160^1^	0.031^*^
	Yes	84 (16)	16 (19.0)	36 (42.9)	16 (19.0)	16 (19.0)	81.40		
PAL	Low	304 (58.0)	104 (34.2)	92 (30.3)	24 (7.9)	84 (27.6)	74.97	12.270^2^	0.002^*^
	Medium	176 (33.6)	96 (54.5)	52 (29.5)	24 (13.6)	4 (2.3)	55.77		
	High	44 (8.4)	32 (72.7)	8 (18.2)	4 (9.1)	0 (0.0)	44.91		

1 Wilcoxon rank-sum test, Z value.

2 Kruskal-Walls test, H value.

^*^The difference was statistically significant.

### 3.2 Univariate analysis of LS in Chinese geriatric cancer survivors

Univariate analyses were executed, designating LS gradation as the dependent variable, while variables from the case report form served as the independent parameters. Determinants such as exercise habits, former occupation type, the presence of tumor metastasis or recurrence, visual or somatosensory abnormalities, and PAL emerged as influential determinants in LS gradation (*p* < 0.05). As delineated in Table 1, subjects who reported an absence of regular exercise, a history of non-physically demanding occupations, tumor metastasis or recurrence, co-existing visual or somatosensory abnormalities, and lower PAL displayed elevated LS gradations.

### 3.3 Multivariate analysis of LS in Chinese geriatric cancer survivors

In this segment, ordinal logistic regression analyses were conducted, incorporating LS gradation as the dependent variable and including only those independent variables that exhibited statistical significance in the univariate analysis. The allocation of these variables is articulated in Table 2. The *p* value of the goodness of fit Hosmer and Lemeshow Test of the included model was >0.05, indicating that the fitting effect was good. The outcomes revealed that the type of former occupation, the occurrence of tumor metastasis, the occurrence of tumor recurrence, and PAL were independent risk determinants exerting a significant influence on LS status among the participants (*p* < 0.05), as substantiated in Table 3.

**Table 2:** Independent variable assignment

Independent variable	Mode of assignment
Exercise habit	None = 0; Yes = 1
Type of former occupation	Non-Physical labor = 0; Physical labor = 1;
Tumor metastasis	None = 0; Yes = 1
Tumor recurrence	None = 0; Yes = 1
Visual abnormality	None = 0; Yes = 1
Somatosensory abnormality	None = 0; Yes = 1
PAL	Low = 0; Medium = 1; High = 2

**Table 3:** Results of ordinal logistic regression analysis of the occurrence and severity of LS in cancer survivors.

Items	Regression coefficient	Standard error	Wald *χ*^2^	*p* value	OR	95% *CI*
Exercise habit	0.080	0.379	0.044	0.834	1.083	−0.664~0.823
Type of former occupation	−0.762	0.361	4.456	0.035*	0.467	−1.470~−0.055
Tumor metastasis	−0.906	0.371	5.947	0.015*	0.404	−1.634~−0.178
Tumor recurrence	−1.075	0.483	4.963	0.026*	0.341	−2.021~−0.129
Visual abnormality	−0.916	0.645	2.020	0.155	0.400	−2.180~0.347
Somatosensory abnormality	−0.074	0.508	0.021	0.884	0.929	−1.069~0.921
PAL (Take the Low level as the reference)						
Medium	1.971	0.762	6.691	0.010*	7.178	0.478~3.464
High	0.685	0.784	0.762	0.383	1.984	−0.853~2.222

*The difference was statistically significant.

## 4 Discussion

### 4.1 The occurrence rate of LS in Chinese geriatric cancer survivors is high

A large group of cancer survivors already exists in China, most of them are older adult. Due to the complexity and specificity of the coexistence of multiple diseases in geriatric cancer survivors, they not only have to bear the discomfort caused by the coexistence of multiple diseases, but also have to bear the physical damage and psychological pressure caused by the former cancer treatment. In the next few decades, this group will expand dramatically with the change of social life pattern and the development of medical technology.

In recent years, a cadre of policy initiatives aimed at promoting comprehensive health engagement has been promulgated by the Chinese Government (17). China is contending with an escalating demographic shift characterized by an aging population (18, 19). Against the backdrop of these dual societal imperatives, there is an intensified focus on enhancing MF, prolonging the duration of autonomous living, and attenuating the caregiving burden on both families and the broader society (19). LS offers a multidimensional diagnostic framework that encapsulates interactions between the musculoskeletal system, overall physiological functionality, body composition, and systemic health metrics. This integrative approach affords a nuanced understanding of functional decline and associated risks, thereby creating opportunities for targeted interventions to mitigate or reverse adverse health trajectories (20).

Five hundred and twenty-four Chinese geriatric cancer survivors were rigorously screened for LS, each of them led an independent, self-sufficient lifestyle. A substantial occurrence rate of 55.7% was identified, with further subclassifications indicating 52.1% at LS-1, 17.8% at LS-2, and 30.1% at LS-3 grades. This salient finding implies that even cancer survivors, who ostensibly lead self-sufficient lives, could be on the cusp of experiencing declines in MF. Importantly, our analysis discerned that the occurrence and severity of LS were not uniformly distributed but were predicated upon various variables: adherence to regular exercise, the physicality of previous occupational roles, metastatic status of the tumor, and the existence of visual or somatosensory abnormalities. Moreover, distinct levels of PA were found to exert differential impacts on the occurrence and severity of LS (*p* < 0.05). Ordinal logistic regression further elucidated survivors who had previously engaged in non-physically demanding occupation, had experienced tumor metastasis or recurrence, and exhibited lower levels of PA manifested a greater propensity for developing LS, often of a more severe grade.

In a parallel context within the East Asian geographical and cultural milieu, Japanese researcher reported an exceptionally high occurrence of LS among 176 cancer survivors, with prevalence rates as follows: LS-1 at 33.5%, LS-2 at 21.6%, and LS-3 at 40.9% (11). Our study revealed a lower occurrence of LS in Chinese geriatric cancer survivors when juxtaposed against their Japanese counterparts. In terms of LS grading, a greater proportion of Chinese geriatric cancer survivors were categorized into LS-1, while fewer populated the remaining severity grades, as compared to the Japanese cohort. This suggests that, while still concerning, the overall occurrence and severity of LS appear to be less pronounced in Chinese geriatric cancer survivors. Two potential contributing determinants warrant mention: ① Sample size in the current study was modest, and it consisted exclusively of cancer survivors who were fully self-sufficient and in relatively stable health; ② Study parameters restricted participation to those survivors who had concluded their primary course of treatment. Therefore, the absence of ongoing therapeutic interventions such as surgical procedures, chemotherapy, or radiation therapy likely renders this sub-population in a comparatively stable health state.

Notwithstanding these observations, it is imperative to note that the prevalence of LS among Chinese geriatric cancer survivors remains significantly elevated when contrasted with general populations that are not afflicted with substantial comorbidities (21–24). This phenomenon may be attributable to a gamut of determinants: direct sequelae like pain, paralysis, and tumor-induced fractures; as well as indirect repercussions such as protein catabolism, augmented inflammatory response, metabolic dysregulation in skeletal muscle, and overall systemic impairment stemming from tumor metastasis and attendant treatments (25, 26). Given the elevated occurrence rates substantiated by this research, there is an unequivocal need for heightened clinical attention to LS in the realm of cancer survivorship care.

### 4.2 Analysis of the risk determinants of the occurrence of LS in geriatric cancer survivors

Prevailing literature has indicated an age-related escalation in the occurrence of LS, with a notably higher prevalence in females relative to males (24–26). Though the current investigation aligned directionally with these observations, indicating elevated LS occurrence in subjects with advanced age categories and a marginally higher occurrence among females, these variations failed to reach statistical significance (*p* > 0.05). Two possible explanations merit consideration: ① Modest sample size imposes limitations on the study’s inferential power; ② Overbearing influence of the cancer pathology might overshadow subtler age and gender-related effects.

### 4.3 Type of former occupation, PAL are risk determinants for LS in geriatric cancer survivors

The present study ascertained a lower occurrence and reduced severity of LS among subjects who had engaged in physically demanding occupations and those exhibiting elevated PAL (*p* < 0.001). This observation finds consonance with Akinobu’s research, which postulates that sustained PA from a young age mitigates the risk of LS in later years (27). Two plausible mechanisms can account for this: ① Survivors engaged in occupations requiring long-term physical exertion or maintaining high PAL likely possess robust foundational physical health. This enhances their resilience to malignancy and its associated therapeutic regimens, expedites recovery from surgical and radiation-induced sequelae, and endows them with sufficient musculoskeletal and cardiorespiratory resilience to counteract fatigue; ② Chronic engagement in physically demanding work or elevated levels of PA confers adaptive benefits to the musculoskeletal system, thereby reducing both the susceptibility and the severity of LS upon its onset.

A corpus of studies has underlined the diminutive PAL observed among cancer survivors (28, 29). This study corroborates those findings, highlighting an overarching paucity of PA in this demographic. The debilitating impact of tumors manifests in multi-systemic functional impairments, including but not limited to, muscular atrophy, osteoporotic changes, pathological pain states, and degenerative musculoskeletal conditions. Such manifestations inevitably circumscribe physical functionality, engendering mobility challenges or even reluctance to engage in outdoor PA, thereby amplifying the risk and exacerbating the severity of LS (30).

The JOA recommends modest quotidian PA as efficacious, yet minimally invasive, interventions for LS prevention (31). Examples encompass commuting by walking or cycling; favoring staircases over elevators; engaging in stretch exercises during television viewing; opting for longer walks to procure groceries, etc. (27). Cumulative enhancements in daily PAL could serve as a viable strategy to attenuate both the occurrence and severity of LS (24, 32).

### 4.4 Survivors with tumor metastasis and recurrence are more likely to develop LS

Our data elucidate that both the metastatic status and recurrence of tumors function as independent predictors for the emergence of LS in geriatric cancer survivors. Individuals grappling with recurrent or middle-to-late-stage malignancies frequently exhibit extensive dissemination of cancerous cells. Subsequently, such patients are predisposed to an augmented regimen of surgical interventions, chemotherapy, and radiation treatments. These therapeutic modalities, while essential, levy a substantial physiologic toll and engender a cascade of adverse reactions. Notable among these are cachexia associated with chemotherapy, osteoporotic alterations in the context of hormone and steroid therapies, and chemotherapy-induced peripheral neuropathy (11). These adverse sequelae deleteriously amplifying both the occurrence and the severity of LS. This underscores the imperative for healthcare practitioners to judiciously manage disease stability during oncological treatments, while proactively addressing symptomology and forestalling both metastasis and recurrence (12).

## 5 Conclusion

The occurrence of LS among Chinese geriatric cancer survivors remains elevated, implicating that even survivors who ostensibly maintain high ADL are experiencing a decline in MF. By contrast, the majority of hospitalized cancer survivors are in progressive disease states, shouldering a more substantial therapeutic burden. It can be inferred that the overall prevalence and intensity of LS among the broader spectrum of cancer survivors could be more pronounced, meriting heightened vigilance of clinical professionals. From a public health perspective, this underscores the need for population-level screening programs aimed at early identification of LS, particularly in cancer survivors who may not exhibit obvious physical impairments but are at risk of functional decline.

Individuals previously engaged in non-physical labor, those with recurrent or metastatic tumors, and those with diminished PAL are predisposed to a higher occurrence and exacerbated severity of LS. Given these findings, public health strategies should prioritize the early detection of LS through regular screenings in geriatric cancer survivors, alongside the development of comprehensive care models that integrate physical activity interventions, rehabilitation programs, and support services. These measures can help mitigate tumor progression, enhance PAL, and ultimately reduce the public health burden associated with LS in this vulnerable population.

## 6 Limitations

The study encompassed only survivors who had completed primary treatment and were managing an autonomous existence, this resulted in a limited sample size, thereby potentially compromising the external validity and generalizability of the findings. Future research endeavors employing larger cross-sectional methodologies are warranted to further explore the prevalence of LS among cancer survivors. Special care must be taken when conducting studies involving cancer as a whole because cancer patients are a heterogeneous population and selection bias may occur in the study population. More LS investigations of geriatric patients with specific tumor types and stages are needed thereafter.

In addition, malabsorption may exacerbate or worsen LS by resulting in deficiency of calcium, vitamin D, and other nutrients vital to the health of bones and muscles. Thus, diet, nutrition and malabsorption will be taken into account in future large sample surveys.

## Data Availability

The raw data supporting the conclusions of this article will be made available by the authors, without undue reservation.
